# A rare case of primary solitary endobronchial plasmacytoma

**DOI:** 10.1111/1759-7714.13853

**Published:** 2021-01-27

**Authors:** Jong Il Park, Yin Young Lee, Seok Soo Lee, June Hong Ahn

**Affiliations:** ^1^ Department of Internal Medicine, College of Medicine Yeungnam University, Yeungnam University Medical Center Daegu South Korea; ^2^ Department of Thoracic and Cardiovascular Surgery, College of Medicine Yeungnam University, Yeungnam University Medical Center Daegu South Korea; ^3^ Division of Pulmonology and Allergy, Department of Internal Medicine, College of Medicine Yeungnam University and Regional Center for Respiratory Diseases, Yeungnam University Medical Center Daegu South Korea

**Keywords:** endobronchial, extramedullary, plasmacytoma, radiation therapy

## Abstract

Extramedullary plasmacytoma (EMP) is a rare plasma cell tumor involving the organs but without bone marrow involvement or the characteristics of multiple myeloma. Primary solitary endobronchial plasmacytoma is extremely rare. Here we present the case of an 86‐year‐old male ex‐smoker who visited our outpatient clinic for an endobronchial mass in the left upper lobe of the lung. Fiberoptic bronchoscopy revealed a protruding mass in the left upper lobar bronchus; based on the bronchoscopic biopsy findings, a primary solitary endobronchial plasmacytoma was diagnosed. After radiation therapy the patient was well and 6 months after treatment showed no evidence of disease recurrence. Extramedullary plasmacytoma should be considered in the differential diagnosis of an endobronchial mass.

## Graphical abstract

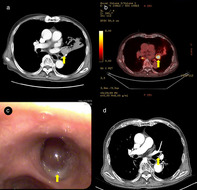

## INTRODUCTION

Extramedullary plasmacytoma (EMP) is a rare plasma cell tumor involving organs outside the bone marrow. Primary pulmonary plasmacytoma (PPP) is a rare variant of EMP^1^ and includes the extremely rare solitary endobronchial plasmacytoma, described in only a few reports.^2^, ^3^, ^4^ Here we present a rare case of solitary endobronchial plasmacytoma without systemic involvement or the characteristics of multiple myeloma.

## CASE REPORT

**FIGURE 1:**
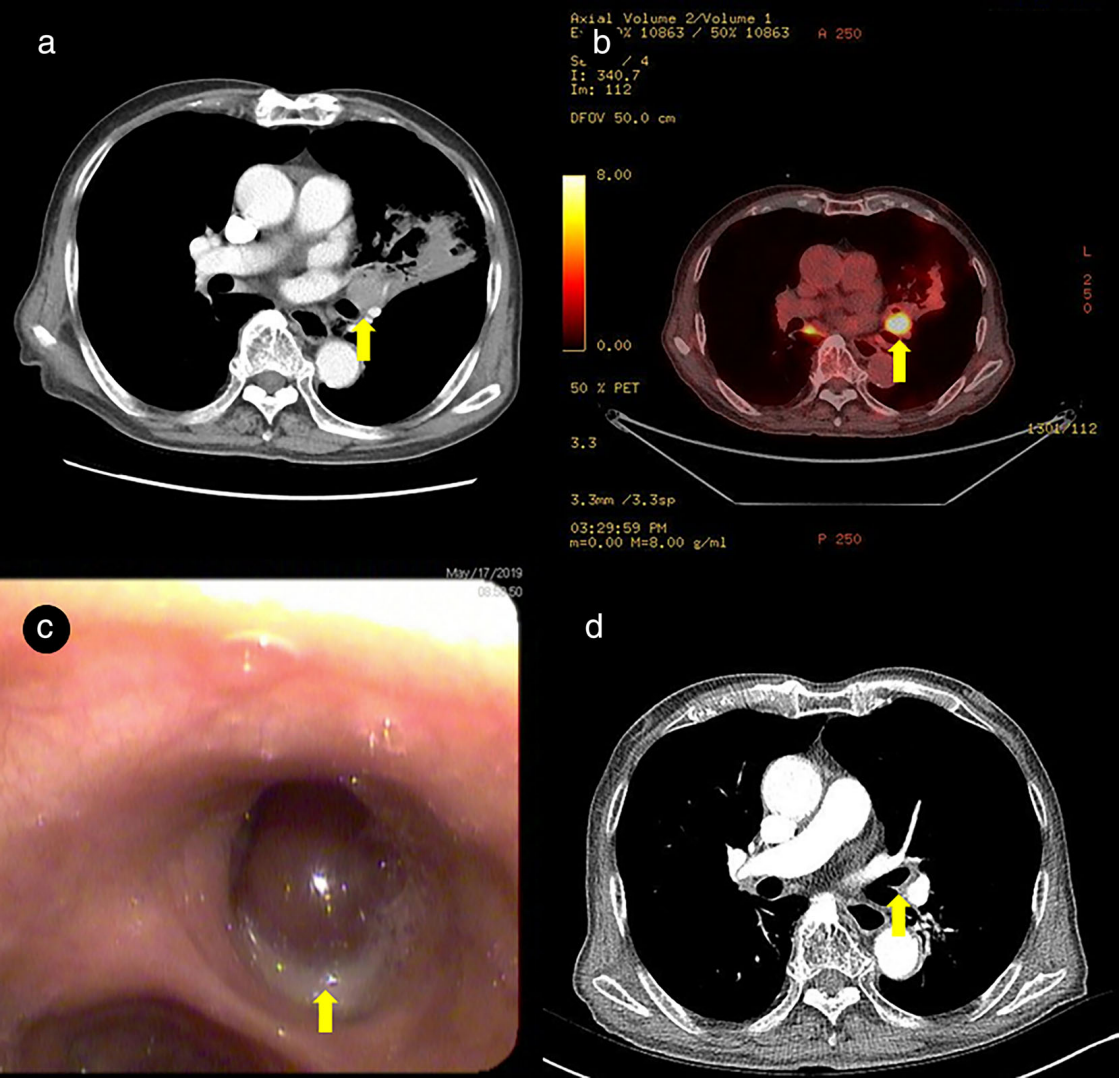
(a) Chest computed tomography (CT) revealed a mass obstructing the left upper lobar bronchus and obstructive pneumonitis. (b) Positron emission tomography (PET) CT revealed a 2.5‐cm fluorodeoxyglucose‐avid nodule involving the left upper lobar bronchus and distal pneumonitis. (c) Fiberoptic bronchoscopy revealed a protruding mass in the left upper lobar bronchus. (d) Chest CT revealed marked improvement of a mass obstructing the left upper lobar bronchus and obstructive pneumonitis after radiotherapy

**FIGURE 2:**
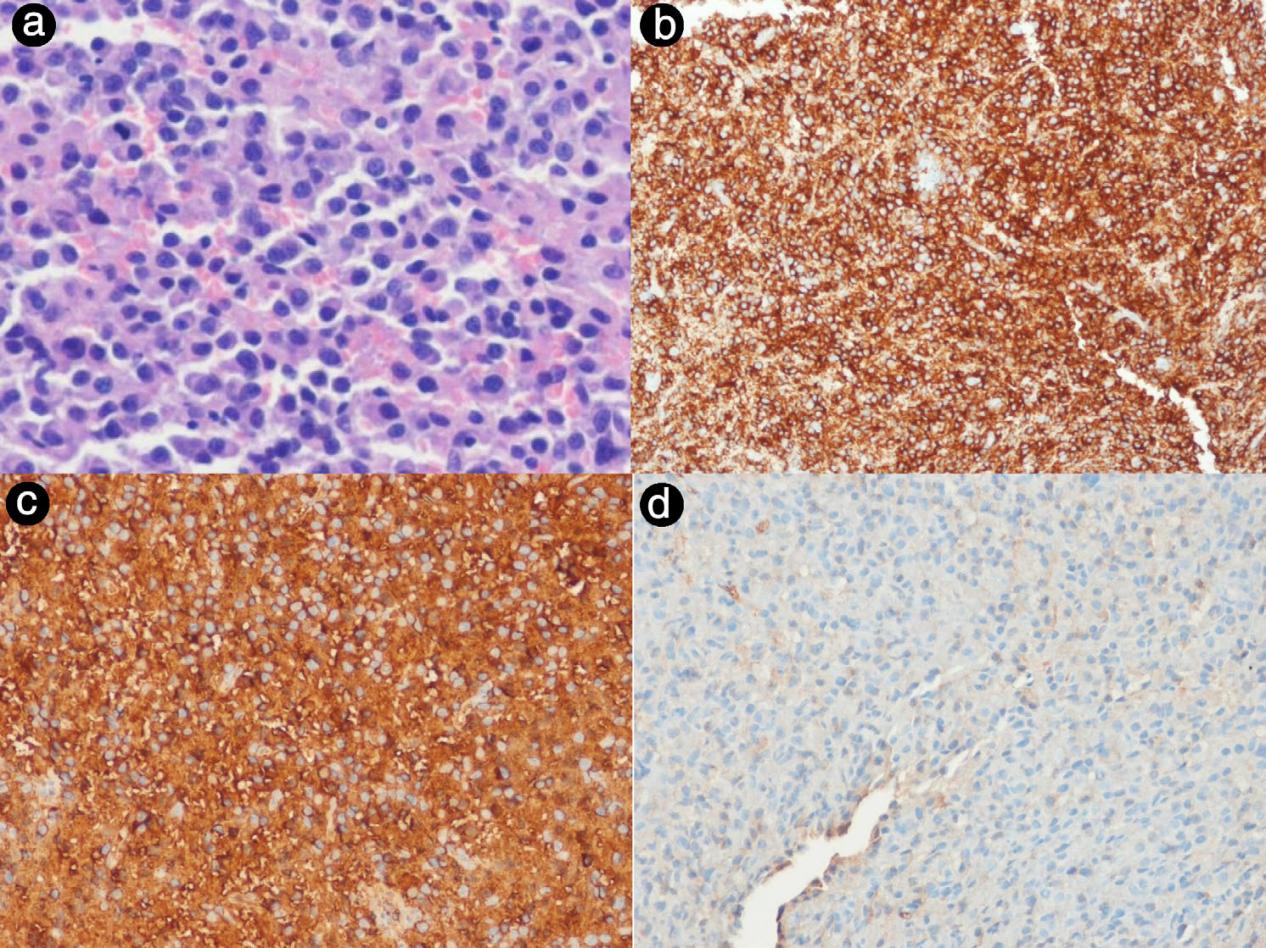
(a) Atypical tumor cells in the mass (H&E, ×200). (b) Immunohistochemical analysis shows CD38 positivity (×200). Staining with kappa (c) and lambda (d) antigens reveals kappa light chain positivity (×200)

An 86‐year‐old male ex‐smoker visited our outpatient clinic complaining of productive cough and dyspnea for a duration of 4 weeks. His blood tests, including complete blood counts, liver and renal function tests, and total protein, albumin, calcium, and cyfra 21‐1 levels, were within normal ranges. Contrast enhanced chest computed tomography (CT) revealed both a mass obstructing the left upper lobar bronchus and obstructive pneumonitis (Figure 1(a)). Positron emission tomography (PET) CT revealed both a 2.5‐cm fluorodeoxyglucose‐avid nodule involving the left upper lobar bronchus and distal pneumonitis (Figure 1(b)). On fiberoptic bronchoscopy, a protruding mass in the left upper lobar bronchus was detected (Figure 1(c)). Bronchoscopic biopsy (PENTAX EB‐1570K video bronchoscope, Olympus single‐use FB‐231D biopsy forcep) was performed without any complications and six pieces of endobronchial tissue were obtained. Biopsy revealed atypical tumor cells with an abundant basophilic granular cytoplasm and eccentrically located round nuclei. Staining for CD38 yielded positive results, and staining for kappa and lambda antigens revealed kappa light chain positivity (Figure 2).

The patient's serum and urine protein electrophoresis findings were normal. The skeletal bone examination was also normal. The bone marrow biopsy revealed a normocellular pattern with no increase in plasma cells. Based on these findings, a primary solitary endobronchial plasmacytoma was diagnosed. Given the patient's age and poor performance status, he underwent radiation therapy consisting of 45 Gy administered in 25 fractions over 5 weeks. Three months after radiation therapy, chest CT revealed marked improvement of a mass obstructing the left upper lobar bronchus and obstructive pneumonitis (Figure 1(d)). The patient responded well to therapy and 6 months after treatment had no evidence of disease recurrence.

This study was a clinical case report and thus did not require Ethics Committee approval. It was performed in compliance with the institutional and national policies concerning research approvals. The patient was informed that clinical details and images concerning his case would be submitted for publication, and consent was provided.

## DISCUSSION

EMP is a rare plasma cell tumor involving organs outside the bone marrow. EMP has two major subtypes, primary (without) and secondary (with prior bone‐marrow involvement).^5^ Pulmonary involvement is usually confined to the lung parenchyma^1^, ^6^, ^7^, ^8^ whereas endobronchial plasmacytoma is a very uncommon variant of EMP, with only a few cases of primary solitary endobronchial plasmacytoma reported thus far.^2^, ^3^, ^4^, ^9^, ^10^, ^11^, ^12^, ^13^ In this case report, we describe a primary solitary endobronchial plasmacytoma detected in an 86‐year‐old male and subsequently treated by radiotherapy.

A systematic case review of EMP revealed that the tumor mainly occurs in males between the ages of 40 and 70. The upper aerodigestive tract is involved in 82.2% of patients and other sites in 17.8%. The treatment options for the former include radiation therapy alone (44.3%), surgery and radiation (26.9%), and surgery alone (21.9%). For an EMP located outside the upper aerodigestive tract, treatment is mostly surgical (55.6%), followed by surgery and radiation (19.8%) and radiation alone (11.1%).^5^ For primary solitary endobronchial plasmacytomas, surgery alone, endoscopic removal, radiation alone, and combined endoscopic removal and radiation have been reported (Table 1).

**TABLE 1:** Literature review of primary solitary endobronchial plasmacytoma

Reference	Age	Sex	Symptom	Location	Diagnostic method	Treatment	Prognosis
Kennedy et al.^11^	17	M	Cough, hemoptysis	Right middle lobar bronchus	Bronchoscopy	Surgical resection	No disease recurrence 2 years after treatment
Kennedy et al.^11^	66	M	Shortness of breath	Trachea	Bronchoscopy	Surgical resection	No disease recurrence 10 months after treatment
Brackett et al.^4^	68	M	Productive cough, dyspnea	Left main bronchus	Bronchoscopy	Endoscopic removal with laser ablation	No disease recurrence
Terzi et al.^12^	65	M	Dyspnea, nonproductive cough	Right main bronchus	Surgery	Surgical resection	No disease recurrence 63 months after surgery
Edelstein et al.^3^	47	M	Wheezing, shortness of breath	Left main bronchus	Bronchoscopy	Endoscopic removal with laser ablation	No disease recurrence 8 months after treatment
Haresh et al.^10^	62	M	Dry cough, hemoptysis	Left main bronchus	Bronchoscopy	Radiation therapy	No disease recurrence 4.5 years after treatment
Park et al.^9^	47	F	Blood‐tinged sputum	Right main bronchus	Bronchoscopy	Endoscopic removal with argon plasma coagulation followed by radiation therapy	Not reported
Zhang et al.^13^	48	M	Dyspnea	Trachea	Bronchoscopy	Endoscopic removal with electrocautery snare followed by radiation therapy	No disease recurrence 6 months after treatment
LeNoir et al.^2^	54	F	Shortness of breath, wheezing	Right main bronchus	Surgery	Surgical resection	No disease recurrence 1 year after treatment
Present case	86	M	Productive cough, dyspnea	Left upper lobar bronchus	Bronchoscopy	Radiation therapy	No disease recurrence 6 months after treatment

According to the literature, 61.1–64.7% of all patients treated for EMP had no recurrence or progression to multiple myeloma; however, in 21.2–22.0% of cases EMP recurred and in 14.1–16.1% it progressed to multiple myeloma.^5^ There is one case report of secondary endobronchial plasmacytoma in patients with a history of multiple myeloma, but there have been no reports of primary endobronchial plasmacytoma progression to multiple myeloma. However, given the relatively high prevalence of EMP recurrence or progression to multiple myeloma in EMP patients other than primary endobronchial plasmacytoma, patients should be carefully monitored.

Our case indicates that primary endobronchial plasmacytoma, a rare presentation of EMP, should be considered in the differential diagnosis of an endobronchial mass. Radiological studies and optimal pathological evaluation will confirm the diagnosis. Treatment strategy will depend on the patient's condition and the extent of the disease. Radiotherapy is a valid option in elderly patients with a tumor located at the level of the lobar or sublobar bronchus, as in our patient. Although there have been no reports of primary endobronchial plasmacytoma progressing to multiple myeloma, patients should be carefully monitored, given the possible progression of EMP.

## CONFLICT OF INTEREST

The authors have no conflicts of interest to declare.
